# Poly(Styrene-Co-Maleic Acid)-Conjugated 6-Aminofluorescein and Rhodamine Micelle as Macromolecular Fluorescent Probes for Micro-Tumors Detection and Imaging

**DOI:** 10.3390/jpm12101650

**Published:** 2022-10-04

**Authors:** Gahininath Y. Bharate, Haibo Qin, Jun Fang

**Affiliations:** 1 Admatius Innovations Pvt. Ltd., G.O. Squre, S.NO. 249/250, Office No. 207, Wakad, Pune 411057, India; 2 SKE Labs, Survery No. 7/1, Jai Malhar Nagar, Thergaon, Pune 411033, India; 3 Laboratory of Microbiology and Oncology, Faculty of Pharmaceutical Sciences, Sojo University, Kumamoto 860-0082, Japan

**Keywords:** fluorescent probes, polymer-dye conjugates, EPR effect, photodynamic diagnosis, tumor imaging

## Abstract

Styrene-co-maleic acid (SMA) copolymer was evaluated as a polymer platform to conjugate with two fluorescent dyes, i.e., 6-aminofluorescein (AF) and Rhodamine (Rho); which spontaneously self-assembles in an aqueous medium and forms a micelle through a non-covalent interaction. These SMA-dye conjugates showed the nanosized micelle formation through dynamic light scattering (DLS) with discrete distributions having mean particle sizes of 135.3 nm, and 190.9 nm for SMA-AF, and SMA-Rho, respectively. The apparent molecular weight of the micelle was evaluated using Sephadex G-100 gel chromatography and it was found that the 49.3 kDa, and 28.7 kDa for SMA-AF, and SMA-Rho, respectively. Moreover, the biodistribution study showed the selective accumulation of the SMA-dye conjugates in the tumor of mice. Taken together, the SMA-dye conjugated micelles appear in high concentrations in the tumor by utilizing the enhanced permeability and retention (EPR) effect of the tumor-targeted delivery. These results indicate that SMA-dye conjugates have the advanced potential as macromolecular fluorescent probes for microtumor imaging by means of a photodynamic diagnosis.

## 1. Introduction

The technique of tumor imaging through fluorescence sounds promising, but still, the limitations are considerable [1,2,3,4]. Cancer remains the major cause of death and its early detection becomes crucial for delaying or preventing cancer-related deaths; for example, the 5-year survival rate of lung cancer patients diagnosed at an early stage is 55%, whereas the survival rate of patients with a late-stage detection is only 4% [5]. Therefore, the early detection of cancer became very important with respect to the survival rate. Tumor imaging techniques such as magnetic resonance imaging (MRI), computed tomography (CT), and single-photon emission computed tomography (SPECT) provides the anatomical information about the tumor in a noninvasive way and is becoming increasingly important for the early detection of cancer. These imaging techniques, however, have limitations such as the limited resolution for visualization, the dissection of a tumor, or the metastasis detection [6].

Over the past few decades, the development of imaging techniques and contrast agents has improved the clinical relevance. Fluorescence imaging has more sensitivity and a superior resolution for the imaging of small tumor nodules compared with other techniques, such as CT scan, MRI, or other scanning techniques [7]. Tumor imaging with fluorescence contrast agents, such as methylene blue, and indocyanine green (ICG) is much more useful in the detection of small metastases that cannot be detected through the preoperative CT, MRI, or intraoperative ultrasonography [8,9]. Moreover, the fluorescent dyes were not originally designed for cancer imaging and their tumor accumulation and selectivity largely depend on the administration route. The fluorescence imaging systems can be integrated with minimally invasive surgical techniques, such as endoscopy, laparoscopic, thoracoscopic, and robot-assisted surgery, in which micro-tumor detection may be possible. Traditional imaging techniques such as radiography, CT, and MRI are difficult to integrate into operating theaters [3], and more importantly, these instruments cannot reliably communicate real-time feedback to the surgeon [7,8,9].

Many companies, enterprises, and research institutes have developed near-infrared (NIR-II) imaging systems. In a large animal model, NIR-II imaging was compared with ICG imaging using NIR-II instruments and a surgical microscope [10,11,12]. A NIR-II imaging system with a camera-mounted surgical microscope gives higher-resolution images with reduced background signals. Integrated imaging systems with visible and near IR multispectral systems, cover a range over 400 nm to 1700 nm [13]. Using this system, multispectral imaging studies in liver cancer patients, injected with ICG, have been carried out; the data demonstrated the usefulness of image-guided surgeries in clinical applications that integrated NIR-I/II imaging with fluorescence probes. NIR-II imaging systems have also benefitted fluorescence endoscopy approaches, by which colorectal cancer could be detected using simultaneous white light and NIR-II fluorescence by a NIR-II fluorescence endoscope in small animal models [14].

In this context, we have developed macromolecular fluorescent probes using polystyrene-co-maleic acid (SMA), which may be delivered to solid tumor tissues more selectively, based on the enhanced permeability and retention (EPR) effect of the macromolecules [15,16,17,18,19,20]. The EPR effect is a unique phenomenon occurring in solid tumors due to the pathophysiological nature of the tumor’s vasculature, where the macromolecules or the high molecular weight drugs accumulate more selectively as compared to low molecular weight drugs [21]. The EPR effect was discovered by Prof. Hiroshi Maeda and Dr. Matsumura [22] in the contest for tumor targeting strategies [23,24]. Macromolecular drug concentration in the tumor can be 10–20 folds higher than that of the blood concentration [18]. Furthermore, a prolonged retention time of the macromolecules or nanoparticles delivered to the tumor, for instance, several days in mice, will be attained in vivo, which is a great contrast to the low molecular weight drugs that will disappear in a few minutes or hours [18,19,20,25]. The EPR effect is not only applied to targeted anticancer therapy [26] but it is also applicable to photodynamic therapy (PDT) [15,27], radiation therapy [28], bacterial therapy of cancer [29], as well as nucleic acid medicine [30].

By using SMA, Maeda et al. successfully developed a SMA-conjugated neocarzinostatin (SMANCS), which was the first polymer-conjugated drug clinically approved in Japan for anticancer therapy [31]. As a nano-platform, SMA has many advantages, and one interesting and important property of SMA is its potential to form micelles in aqueous media [31,32]. Accordingly, many SMA micelles have been developed with various compounds, such as anticancer agents; doxorubicin [33], pirarubicin [34], zinc protoporphyrin [35], taxol, camptothecin, cisplatin [36], aclarubicin [37], etc. All of the SMA micelles showed good tumor accumulation properties based on the EPR effect. Moreover, the SMA-based micelles always show a quick and active intracellular uptake, as evidenced in our previous studies [15,38,39]. This may be due to the amphiphilic nature of SMA; which increases the affinity between SMA and the surface receptor of the cells. The rapid and high intracellular uptake of SMA micelles with photosensitizers (SMA-PS), i.e., SMA micelles of methylene blue (SMA-MB) and rose bengal (SMA-RB) have the advantages to be more effective PDT photosensitizers [15].

Along this line, we report, the synthesis, characterization, and evaluation of the SMA conjugate of the fluorescent probes 6-aminofluorescein (SMA-AF) and rhodamine (SMA-Rho), which form micelles through self-assembly in water solutions. Their in vitro characterization and in vivo properties were further evaluated for microtumor imaging by means of a photodynamic diagnosis.

## 2. Materials and Methods

### 2.1. Materials

Rhodamine 123 {[7-Amino-10-[2-(methoxycarbonyl)phenyl]-2H-xanthene-2-iminium chloride} (Rho), 6-aminofluorescein (AF), 1-ethyl-3-(3-dimethylaminopropyl)carbodiimide (EDAC), and bovine serum albumin (BSA) were purchased from Wako Pure Chemical Industry Osaka, Japan. A SMA anhydride with mean Mw. 1580 was purchased from Kuraray Co. Ltd., Osaka, Japan. An anhydrous SMA polymer obtained from the manufacturer was of a low polydispersity, and it was purified using the purification method as described by Iyer et al. [35]. Other reagents of reagent grade, solvents, and chemicals were purchased also from Wako Pure Chemical Industries (Osaka) and were used without further purification.

### 2.2. Synthesis of the SMA-6-aminofluorescein Conjugate (SMA-AF), and the SMA-rhodamine Conjugate (SMA-Rho)

**Scheme 1:**
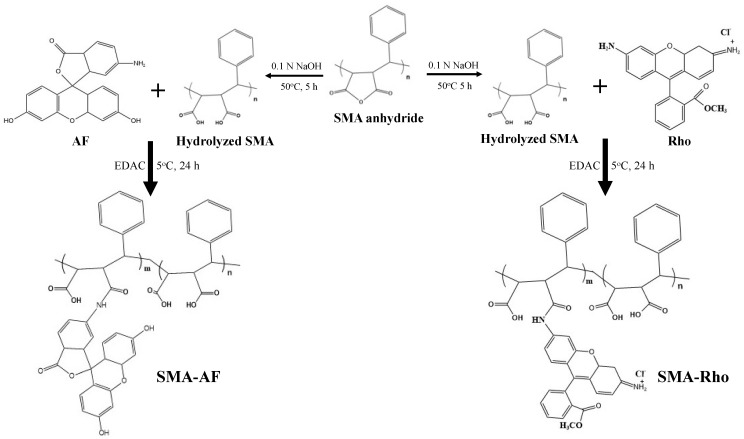
Synthetic representation of the formation of the polystyrene-co-maleic acid-6-aminofluorescein conjugate (SMA-AF) and SMA-rhodamine conjugate (SMA-Rho) by the coupling reaction of polystyrene-co-maleic acid (SMA) with the 6-aminofluorescein (AF) in the presence of 1-ethyl-3-(3-dimethylaminopropyl)carbodiimide (EDAC). SMA has 7 to 8 repeating units. The AF or Rho is conjugated to the ionized carboxyl group of the maleic acid residue of the SMA chain.

As shown in the reaction Scheme 1, the synthesis of the SMA-AF and SMA-Rho conjugates was carried out in a two-step reaction: (1) hydrolysis of SMA, and (2) conjugation of the 6-aminofluorescein to the hydrolyzed SMA. Firstly, the maleic anhydride residue of the SMA polymer was hydrolyzed with the addition of 0.1 N NaOH at 10 mg/mL. The solution was heated at 50 °C and stirred for 24 h until a clear solution was obtained. The hydrolysate was then neutralized with 0.1 M HCl to pH 7.0, followed by dialysis and freeze-drying to obtain the hydrolyzed SMA.

Then, the SMA-dye conjugates were synthesized using the hydrolyzed SMA in the presence of a water-soluble coupling agent EDAC. Briefly, the hydrolyzed SMA (1.5 g, 7.5 mmol of SMA unit, assuming a mean repeating unit of 7–8 per SMA polymer chain) was dissolved in deionized water (50 mL), then, the solution was cooled to 5.0 °C using an ice bath and EDAC (248 mg; 1.597 mmol) pre-dissolved in deionized water (10 mL) and the pH was adjusted to 5.0. Then, the AF (300 mg; 0.86 mmol) or Rho (250 mg; 0.656 mmol) was added in aliquots of 50 mg each time for 30 min. Following the addition of the AF or Rho, the reaction mixture was stirred for another 24 h at room temperature, in the dark. Then, the solvent was evaporated using a rotary evaporator (BUCHI Labortechnik AG, Switzerland) with the temperature maintained at 55 °C. The powder thus obtained was further purified using Sephadex G-100 gel chromatography (GE Healthcare, Chicago, IL, USA) in a column (h 45 cm × Φ 2.5 cm). The eluted 2.5 mL of each fraction was monitored with a UV absorbance at 488.0 nm or 484 nm corresponding to the SMA-AF and the SMA-Rho, respectively. The peak fractions were pooled and lyophilized in order to yield the powder of the SMA-AF and SMA-Rho conjugates, which were subjected to further characterization for the structural determination, including UV/Vis, FTIR spectroscopy, fluorescence spectroscopy, etc.

### 2.3. Dynamic Light Scattering (DLS) and the Zeta Potential

The particle sizes in the aqueous solution of the SMA-AF and the SMA-Rho were determined with a Photal DLS-7000 HLs laser-light scattering spectrophotometer (Otsuka Electronics, Osaka, Japan), equipped with a 10 mW He-Ne (632.5 nm) laser light source. For the DLS measurements, the scattering angle was fixed at 90° and the temperature of the sample was maintained at 25.0 ± 0.01 °C. The samples at the concentration of 1.2 mg/mL were prepared in a 0.01 M sodium phosphate buffer (pH 7.4), which yielded the optimal counts.

### 2.4. UV-Visible Spectroscopy

The UV/visible absorption spectra were recorded on a spectrophotometer (Model UV/Vis U-3900, HITACHI Corp., Tokyo, Japan). The contents of the dyes (loading) in the SMA-AF and SMA-Rho were quantified using standard curves for free dyes in a 0.01 M sodium phosphate buffer (pH 7.5). In brief, the various concentrations of the free AF or Rho (0.02, 0.04, 0.06, 0.08, 0.1, and 0.12 mg/mL) were prepared, and the absorption was measured, by which the graph of the absorption vs. the concentration was plotted and the standard curves were calculated. The SMA-AF or SMA-Rho was dissolved in a 0.01 M sodium phosphate buffer (pH 7.5) at 1 mg/mL, and the maximal absorption corresponding to the AF or Rho was measured, by which the dye content in the SMA-dye conjugated was calculated as the “calculated dye concentration in the SMA-dye solution × 100/1 mg/mL.

### 2.5. Fourier Transforms Infrared (FTIR) Spectroscopy

Fourier transform infrared (FTIR) spectra were recorded on a FT/IR-4200 spectrometer (JASCO Corp., Tokyo, Japan) using KBr pellets.

### 2.6. Fluorescence Spectroscopy

Fluorescence spectra were recorded on a F-2500 spectrometer (Hitachi, Tokyo, Japan). The sample solutions of the SMA-Rho were excited at 495 nm (corresponding to Rho), and the SMA-AF solutions were excited at 480.0 (corresponding to AF).

### 2.7. In Vivo Tissue Distribution of the SMA-AF and SMA-Rho Conjugates: In Vivo Fluorescence Imaging

Male ddY mice o 6 weeks of age (SLC Inc., Shizuoka, Japan) were used in the tissue distribution (in vivo imaging) study of the SMA-AF and SMA-Rho conjugates. The mouse sarcoma S180 cells (2 × 10^6^ cells) that had been maintained in the peritoneal cavity of the ddY mice in an ascetic form were implanted subcutaneously (s.c.) in the dorsal skin of the mice in order to establish a mouse S180 solid tumor model. All animals were housed at 22 ± 10 °C and 55% ± 5% relative humidity with automatic lighting at a 12-h light/dark cycle. All experiments were carried out according to the Laboratory Protocol of Animal Handling, Sojo University, and were approved by the Animal Ethical Committee, Sojo University (No. 2012-P-003, approved on 1 April 2012) and were carried out according to the Guidelines of the Laboratory Protocol of Animal Handling, Sojo University.

At 10–12 days after the tumor inoculation, when the tumor grew to approximately 10 mm in diameter, the SMA-AF and SMA-Rho conjugates that were dissolved in the physiological saline, were injected intravenously (i.v.) at 5 mg/kg (fluorescent dye equivalent). Following a 24 h period, the mice were subjected to in vivo imaging using IVIS XR (Caliper Life Science, Hopkinton, MA, USA). Then, the mice were sacrificed and the tumors, as well as the normal tissues, were collected for ex vivo fluorescence imaging using IVIS XR.

### 2.8. Statistical Analyses

All data were expressed as means ± SD. The data were analyzed using ANOVA followed by the Bonferroni multiple comparison test. A difference was considered statistically significant when *p* < 0.05.

## 3. Results and Discussion

### 3.1. Synthesis and Characterizations of the Polystyrene-Co-Maleic Acid-6-Aminofluorescein (SMA-AF) and the Polystyrene-co-maleic Acid-rhodamine (SMA-Rho) Conjugates

In the present work, we synthesized the SMA-AF and SMA-Rho conjugates, which spontaneously self-assembled and formed micelles in the aqueous medium. The purified SMA, having a mean molecular mass of 1580, was used to synthesize the SMA-AF and SMA-Rho conjugates.

**Figure 1:**
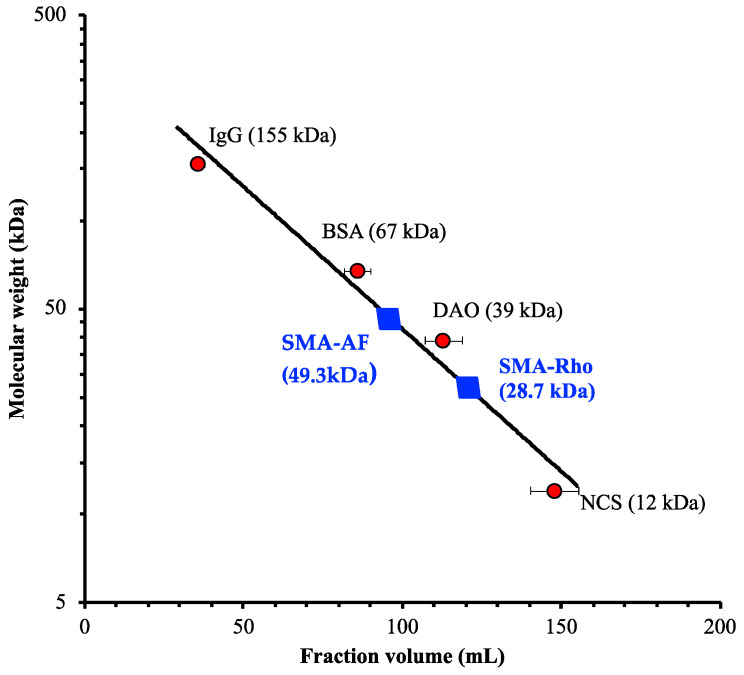
Sephadex superfine G-100 gel chromatography of the SMA-AF and the SMA-Rho conjugates. Sephadex size-exclusion gel chromatography of the SMA-dye conjugates was carried out with various known proteins of different molecular weights (NCS, DAO, BSA, IgG) as reference standard; 2.5 mL fraction was collected per tube and a 0.1 M sodium phosphate (pH 7.4) buffer was used as an eluent. Experiments were repeated three times, and data are mean ± standard deviation (SD).

Based on the UV/visible absorption spectra and the standard curves of the AF and Rho, the loading of the AF and Rho in the SMA-AF and SMA-Rho was calculated to be 6.36% w/w and 8.72% w/w, respectively. Accordingly, the mean molecular weights based on the chemical formula of both SMA-AF and SMA-Rho conjugates were about 1.93 kDa. However, chromatography using a Sephadex G-100 superfine high-resolution gel showed large apparent molecular weights of 49.3 kDa and 28.7 kDa, respectively (Figure 1; Table 1), based on the standard marker proteins of different molecular weights, i.e., neocarzinostatin (NCS), 14 kDa; D-amino acid oxidase (DAO), 39 kDa; bovine serum albumin (BSA), 67 kDa; ImmunoglobilinG (IgG), 155 kDa. These results indicate that, irrespective of the molecular weight of the SMA chain (1.92 kDa), the SMA-dye conjugates self-associate to form the supramolecular assemblies in the aqueous medium, similar to the findings reported earlier for other polymers, such as PEG and other polymer drug conjugates [40,41,42]. Similar results were also seen in our previous studies which the PEG-conjugated 4-Amino-6-hydroxypyrazolo [3,4-d]pyrimidine (AHPP) [43] and the SMA-conjugated AHPP showed large molecular weights of about 116 kDa and 67 kDa, respectively, using Sephadex gel chromatography [44,45]. It is anticipated that these macromolecular conjugates are capable of utilizing the EPR effect for the tumor-selective delivery [22,46].

### 3.2. Behaviors of the SMA-AF and SMA-Rho Conjugates in Aqueous Solutions, as Analyzed Using Dynamic Light Scattering (DLS)

**Figure 2:**
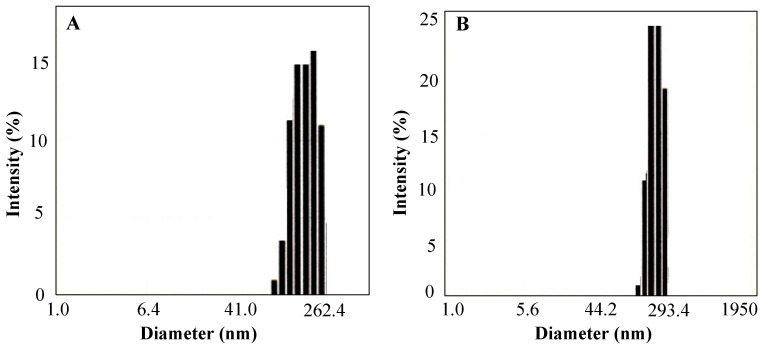
Dynamic light scattering (DLS) of the SMA-AF (**A**,**B**) SMA-Rho. The samples for DLS were prepared in a 0.01 M sodium phosphate buffer (pH 7.4) at 1.2 mg/mL. The average particle size of the micelles was 135.3 nm and 190.9 nm for the SMA-AF conjugate and the SMA-Rho conjugate, respectively. The plot of the Ls Int. Distribution vs. the diameter was shown.

The self-association of the polymeric micellar structure of the SMA-AF and SMA-Rho conjugates was further elaborated through DLS studies. The SMA-AF and SMA-Rho conjugates exhibited a mean particle size of 135.3 nm and 190.9 nm, respectively (Figure 2; Table 1). These results are similar to and in line with the previously reported SMA-AHPP conjugate [44], SMA-photosensitizers [15], and PEGylated zinc protoporphyrin [35] in our laboratory, which behave similarly in the aqueous medium in a similar fashion, by forming self-assembled associations. This result indicates that the SMA-AF and SMA-Rho conjugates self-assembled probably through a non-ionic hydrophobic interaction, to form the micelles. In addition, considering that the BSA (67 kDa) shows a size of 5–10 nm through DLS, the sizes of the SMA-dye conjugates measured through DLS were much larger than those obtained using Sephadex G-100 gel chromatography. This is probably due to the interaction between the Sephadex gel with the SMA molecules, which resulted in an apparently smaller size, and may thus not reflect the real size of the SMA-AF and SMA-Rho conjugates in water solutions.

### 3.3. UV/vis and FTIR Characterization of the SMA-AF and SMA-Rho Conjugates

The Sephadex-G100 purified SMA-AF and SMA-Rho conjugates were further analyzed using UV spectrophotometry. As shown in Table 2, the SMA-AF and SMA-Rho conjugates have maximal absorption peaks at 488.0 nm, and 494.0 nm, and the emission wavelength of 517.0 nm and 525.0 nm, respectively. Thus, the quantification of the conjugated dyes (AF and Rho) in SMA was carried out by plotting a standard curve of free dyes in a 0.01 M phosphate buffer at the physiological pH (pH 7.5). The loading of the incorporated dyes and the characterization of the SMA-dye conjugates were summarized in Table 1.

**Figure 3:**
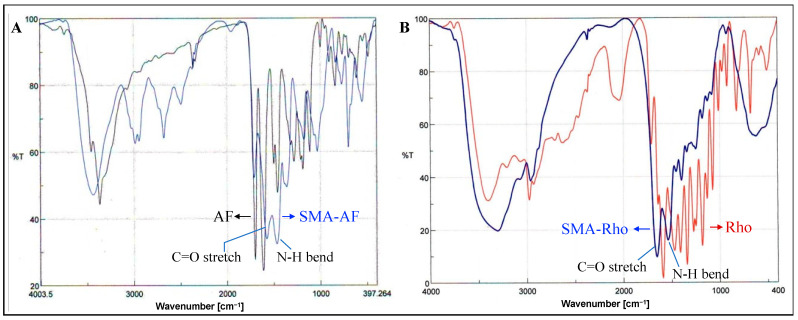
Fourier transform infrared (FTIR) spectra of the SMA-AF vs. the free 6-aminofluorescein (**A**) and the SMA-Rho vs. the free Rhodamine (**B**).

Further, the conjugation between the carboxylic group of the maleic acid residue of the SMA copolymer and the amino group of the AF by the amide linkage was confirmed using FTIR spectroscopy. The FTIR spectrum of the SMA-AF clearly showed the peaks corresponding to the amide—I (1654 cm^−1^ strong C=O stretching) and amide—II (1537 cm^−1^ corresponding to N-H bending) frequencies (Figure 3); whereas, the polymer of the SMA showed anhydride stretching frequencies at 1837 cm^−1^ and 1760 cm^−1^ (data not shown), which disappears due to the opening of an anhydride ring during the conjugation and the hydrolysis reaction (Figure 3). Furthermore, a similar FTIR interpretation as the SMA-AF for the conjugation of the SMA copolymer and the amino group of Rho, was confirmed through amide linkage stretching in the FTIR.

### 3.4. In Vivo Tissue Distribution of the SMA-AF Conjugate: In Vivo Fluorescence Imaging

**Figure 4:**
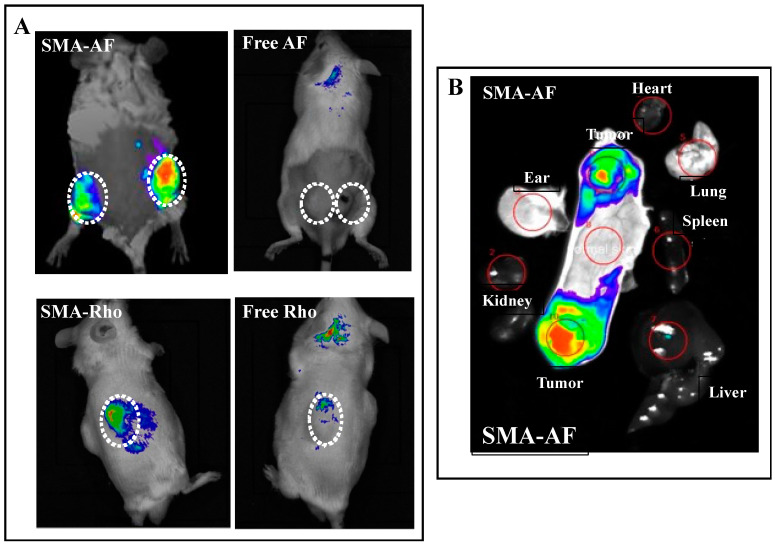
In vivo imaging of the SMA-AF and SMA-Rho in a S180 solid tumor model. SMA-AF or SMA-Rho, as well as the responding free AF or free Rho, were injected i. v. into the ddY mice bearing S180 solid tumors Following a period of 24 h, the mice were subjected to in vivo imaging using the IVIS imaging system. Dashed circles indicate the position of the tumors (**A**). In mice treated with the SMA-AF, after in vivo imaging, the mice were sacrificed and the tumors, as well as the normal tissues and organs, e.g., the heart, liver, kidney, spleen, lung, and ear, were collected, and the fluorescence intensity in each tissue was detected and compared using the IVIS imaging system (**B**).

Finally, in a preliminary study, we performed in vivo imaging using the SMA-AF as compared to the free AF. As shown in Figure 4, the SMA-AF conjugates showed the tumor-selective accumulation after the i.v. injection in the ddY mice bearing S180 solid tumors. Compared to the free AF, for which no apparent fluorescence was detected at 24 h after the i.v. injection, an intense fluorescence was detected in the tumors (Figure 4A). When we resected the tumors and the normal tissues and compared the fluorescence intensity in each tissue, we found that a strong fluorescence was only detected in the tumors (Figure 4B), suggesting that the SMA-AF directly accumulated in the tumors after the i.v. administration. Similar results were also seen for the SMA-Rho, compared to the free Rho that showed almost no distribution in the tumor at 24 h after the i.v. injection. The SMA-Rho exhibited a much more extensive tumor-selective fluorescence (Figure 4A). This tumor-selective distribution of The SMA-AF and SMA-Rho is mostly attributed to the EPR effect. We thus anticipate the application of the SMA conjugated fluorescence dyes (e.g., SMA-AF and SMA-Rho) for the detection of tumors, especially small tumor nodules, such as metastatic tumors and disseminated tumors, by taking advantage of the EPR effect-based tumor accumulation and the high sensitivity of the fluorescence detection. However, it should be noted that the mouse subcutaneous S180 solid tumor model was used in the study, which is known to exhibit a good EPR effect [32]. Though this model is good for screening antitumor nanomedicines, it could not ensure the effectiveness of the nanomedicines in other tumor models, especially human cancers. It is now well-recognized that some mouse tumor models, such as the pancreatic cancer model, as well as many other human cancers, especially those advanced staged tumors, usually show a poor EPR effect because of the poor or occluded tumor blood flow, thus the improvement of the tumor blood flow has become an essential issue for achieving a satisfying therapeutic effect of nanomedicines, based on the EPR effect [32]. In this context, further investigations using tumor models mimicking human cancers, such as orthotopic models and carcinogen-induced carcinogenesis models, are warranted, which will be carried out in our future study.

The macromolecular micellar property of the SMA-AF renders its tumor-targeted accumulation, based on the EPR effect. The DLS and Sephadex G-100 gel chromatography studies of the SMA-dyes indicated that the SMA-AF, as well as the SMA-Rho, form the supramolecular self-assembled association to behave as micelles in the aqueous medium; the critical micellar concentration (CMC) of both the SMA-AF and SMA-Rho was found to be about 0.25 mg/mL; therefore, above the CMC level, the SMA-dye conjugates will behave as macromolecules in blood circulation, thus exhibiting a prolonged circulation time. In addition, due to their macromolecular nature, the SMA-dye conjugates may accumulate in the site of interest, i.e., solid tumors as well as inflammatory tissues, by taking advantage of the EPR effect [31], thus demonstrating their pharmacological effect more selectively, and greatly decreasing the potential side effects. The concept of macromolecular drugs or nanomedicines is now widely applied in various clinical fields. For example, native interferon therapy for hepatitis has been almost replaced by PEG-interferon therapy [39,46].

The direct driving force for the micelle formation process is probably the hydrophobic interaction. The self-assembled associations have also been observed in block copolymer systems, consisting of hydrophilic and hydrophobic chains, termed ‘polymeric micelles’ [42]. The physicochemical and biological importance of these polymeric self-assembled micelles and their application in anticancer drug carriers, as well as photodynamic therapy has been extensively studied by many researchers [47,48]. Herewith, the SMA-dye conjugates or SMA-fluorescent-probes described in this study, by benefiting their nano-micelle formation, accumulate in the tumor through the EPR effect and thus exhibit the potential for detection/diagnosis of solid tumors with a high sensitivity.

## 4. Conclusions

In conclusion, here we successfully synthesized and evaluated the SMA-AF and SMA-Rho conjugates. The conjugation with the SMA results in the nano-micelle formulation of the fluorescence dyes in the aqueous solution, which could accumulate in tumor tissues much more selectively, based on the EPR effect. This macromolecular fluorescence imaging will help surgeons to locate and navigate the position of specific micro-tumor nodules and become a guide for the surgery of micro-tumors in a real-time manner. Specifically, the modality labels the tissue at the molecular/cellular level, thus facilitating a superior resolution and sensitivity when compared to the pre-operative radiolabeled imaging techniques. We thus anticipate the application of SMA-dye conjugates for the early diagnosis of micro-tumors, and we warrant further investigations with this platform using SMA-fluorescent probe technology.

## Figures and Tables

**Table 1:** Properties and characterization of the SMA-AF and SMA-Rho conjugates.

^a^ SMA Dyes	^b^ Apparent Molecular Weight (kDa)	^c^ Particle Size (nm)	^d^ Dye Content (%W/W)	^e^ Surface Charge (ζ, mV)
SMA-AF	49.3	135.3	6.36	−46.03
SMA-Rho	28.7	190.9	8.72	−43.03

^a^ SMA-AF and SMA-Rho conjugate as SMA fluorescent dyes. ^b^ The hydrodynamic size of the nano micelles in the aqueous medium was determined using Sephadex G-100 gel chromatography. ^c^ Particle sizes of the SMA- dyes were determined using dynamic light scattering (DLS) at the concentration of 1.2 mg/mL in a phosphate buffer (pH 7.5). ^d^ Contents of the fluorescent dyes (AF and Rho) in the SMA-dye conjugates were measured using UV absorption spectroscopy with standard curves of the free dyes. ^e^ Surface charge on the SMA-AF and SMA-Rho conjugates were determined using the zeta potential and a particle size analyzer, ELSZ-2 Otsuka, Japan, in which the SMA-dyes were prepared at 1.2 mg/mL in deionized water.

**Table 2:** The absorption and emission properties of the SMA-AF and SMA-Rho conjugates.

^a^ SMA-Dyes	^b^ Absorption (λex, nm)	Abs. Max. (λmax, nm)	^c^ Emission (λex, nm)	Emission Max. (λem, nm)
SMA-AF	405–495	488.0	465–570	517.0
SMA-Rho	430–590	494.0	475–620	525.0

^a^ SMA-AF and SMA-Rho conjugates. ^b^ The UV/visible absorption spectra were recorded on a spectrophotometer (Model UV/Vis U-3900, HITACHI Corp., Tokyo, Japan). ^c^ Fluorescence spectra were recorded on a F-2500 spectrometer (Hitachi, Tokyo).

## Data Availability

The data presented in this study are available upon request from the corresponding author.
